# Acute Dysentery, Treatment

**Published:** 1875-07

**Authors:** 


TREATMENT OF ACUTE DYSENTERY.—Dr. J. R. Cushing, of Texas, suggests, in *Chicago Medical Journal*, the following:

℞.—Magnesiæ Sulphatis . . . . . . ℥j.

Tinct. Opii, . . . . . . . . ʒj.

Aquæ, . . . . . . . . . ℥vj.

M. Sig. Tablespoonful every half hour until it acts. Cold cloths to perineum, and cloth saturated with the following recipe kept constantly applied to the abdomen.

℞.—Aquæ Ammoniæ.

Ol. Sassafras, . . . . . . aa. ℥ss.

Tinct. Opii.

Tinct. Arnicæ, . . . . . . . aa. ʒj.

Ol. Olivæ.

Ol. Kerosene, . . . . . . . aa. ℥ij.

Misce.

After the action of the salts, substitute the following formula:

℞.—Creasoti . . . . . . . . gtt. xx.

Acidi Acetici, . . . . . . . gtt. xl.

Morph. Sulphatis, . . . . . . gr. ij.

Aquæ, . . . . . . . . ℥ij.

Misce.

Sig. Teaspoonful every two hours until relieved.

